# Direct gambling marketing, direct harm: A randomised experiment

**DOI:** 10.1111/add.70369

**Published:** 2026-03-18

**Authors:** Matthew Rockloff, Matthew Browne, Nerilee Hing, Alex M. T. Russell, Vijay Rawat, Philip Newall

**Affiliations:** ^1^ Experimental Gambling Research Laboratory, School of Health, Medical and Applied Sciences Central Queensland University Bundaberg Queensland Australia; ^2^ Experimental Gambling Research Laboratory, School of Health, Medical and Applied Sciences CQUniversity Sydney New South Wales Australia; ^3^ Experimental Gambling Research Laboratory, School of Health, Medical and Applied Sciences CQUniversity Melbourne Victoria Australia; ^4^ School of Psychology Deakin University Burwood Victoria Australia; ^5^ School of Psychological Science University of Bristol Bristol UK

**Keywords:** betting behaviour, direct marketing, ecological momentary assessment, experimental study, gambling harm, harm reduction, randomised controlled trial

## Abstract

**Background and aims:**

Whether gambling marketing has a causal effect on harm is of regulatory interest. Direct marketing offers (emails, push notifications and text messages) are frequently received by people with active gambling accounts, but they can opt out. This study aimed to test whether opting out of direct marketing reduces betting and short‐term gambling harms in a real‐world gambling environment.

**Design:**

Stratified randomised field experiment with a between‐participants design, embedded in a 14‐day ecological momentary assessment (EMA).

**Setting:**

Nationwide, Australia (July–August 2023).

**Participants:**

Participants (*n* = 227; 61.7% men; mean age = 45), 52.0% of whom scored in the moderate‐risk or problem range on the Problem Gambling Severity Index (PGSI), were regular Australian sports and race bettors recruited from online panels who agreed in principle to opt out of receiving direct marketing from all wagering operators with whom they held accounts.

**Intervention:**

Participants were stratified by PGSI risk category, age group and gender and then randomly allocated either to an opt‐out condition (*n* = 96), in which they opted out of direct marketing from their wagering operators and provided proof, or to a control condition (*n* = 131) that continued to receive direct marketing as usual.

**Measurements:**

Seven EMA surveys were administered every 48 hours over a 14‐day period during a high‐volume betting season. Outcomes were self‐reported number of bets placed, gambling expenditure (in Australian dollars, AUD) and short‐term gambling harms in the previous 48 hours, assessed with a 10‐item adapted Short Gambling Harm Screen (SGHS).

**Findings:**

The opt‐out group placed 23% fewer bets [*B* = −0.11, 95% confidence interval (CI) = −0.20, −0.03, *P* = 0.011], spent 39% less money (*B* = −0.53, 95% CI = −0.84, −0.21, *P* = 0.001) and reported 67% fewer short‐term gambling harms (*B* = −0.22, 95% CI = −0.36, −0.07, *P* = 0.004) compared with the controls.

**Conclusions:**

Opting out of receiving direct marketing from wagering operators appears to be associated with statistically significantly fewer bets made, amount spent gambling and short‐term gambling harms.

## INTRODUCTION

Since the COVID‐19 pandemic, the gambling industry in Western markets has experienced sustained growth, largely driven by online and mobile sports betting on smartphones [1, 2]. Direct marketing is a particularly important means for promoting smartphone betting [3, 4, 5, 6, 7], where gambling operators contact customers via emails, text messages, app notifications and phone calls that prompt people to place bets based on special offers or deliver reminders of upcoming sporting events. For context, Syvertsen *et al*. [8] found that individuals with a current or past gambling disorder frequently receive direct marketing messages, often several times a day, through emails, text messages and phone calls. Many individuals described direct marketing as intrusive, personally tailored and difficult to avoid during their attempts to abstain from gambling [8].

### Direct marketing and gambling behaviour

Although much of the existing evidence is correlational, and therefore open to alternative causal explanations, several studies have demonstrated strong associations between direct marketing exposures and betting behaviour. Across qualitative studies, direct marketing offers have been characterised as appealing and beneficial during active gambling, yet aggressive, triggering and harmful during attempts to reduce or abstain from gambling [9, 10, 11, 12, 13]. Exposure to text messages from wagering operators has been shown to be correlated with an increase in both betting and gambling spend within a 24‐hour window [3]. Betting consumption and harm have been found to also increase with each message received from wagering operators, betting tipsters and free betting information services [14]. Moreover, wagering inducements, such as stake‐back offers and bonus bets, are associated with elevated intentions to bet and actual betting [6, 12, 15].

Wagering inducements are also associated with increased impulse betting [16]. Survey respondents have reported that receiving promotional offers, such as through text messages, cause them to make spur‐of‐the‐moment bets [3]. Direct marketing appeals may exacerbate impulsive betting behaviour, particularly among individuals who already exhibit higher levels of trait impulsivity, with cues such as inducements triggering spur‐of‐the‐moment bets [17, 18]. In a sample of 1813 Australian sports bettors, the uptake of wagering inducements has been linked to higher levels of impulse betting amongst higher‐risk gamblers, and trait impulsivity was associated with both harmful gambling and a greater likelihood of betting on impulse [16, 19]. However, these associations may also reflect alternative pathways. For example, operators may send more inducements to people who already bet heavily, or individuals experiencing losses may seek out promotions more actively.

Existing correlational studies cannot speak to whether direct marketing promotions cause people to escalate their betting beyond levels of entertainment or enjoyment, resulting in significant gambling‐related harm. Beyond direct marketing, recent systematic reviews of gambling advertising report only a few experimental or quasi‐experimental studies that manipulate exposure to gambling advertisements, typically short‐term laboratory cue‐exposure designs or natural experiments around changes in advertising volume or regulation, and these generally find that greater exposure increases betting intentions, urges or expenditure [20, 21]. The present study was conceived to improve the quality of evidence regarding these issues by experimentally manipulating exposure to direct marketing in a naturalistic setting using an ecological momentary assessment (EMA).

### Regulatory relevance

An improved understanding of how direct marketing appeals may cause harm is important for consideration in product regulation. Restrictions in marketing have been effective in supporting generational change away from smoking and reducing its attendant harm [22, 23]. The accumulation of causal evidence regarding tobacco marketing has been instrumental in garnering support for restrictions on advertising [24, 25, 26]. Contrastingly, there is less regulatory consensus in gambling. While some European jurisdictions are restricting the legal availability of gambling marketing [27, 28, 29], some Canadian and US states are seeing expanded legal markets and attendant marketing increases [30, 31]. Gambling policymakers often state that the evidence base is insufficient to support widespread restrictions [32]. However, this evidence base remains limited because experimental and quasi‐experimental studies of gambling advertising are still rare and tend to focus on short‐term outcomes or aggregate data, and high‐resolution individual‐level data suitable for causal econometric analyses are scarce. The present research can provide evidence on the effects of direct marketing in naturalistic environments, which can help inform policymakers considering actions on direct marketing, as well as inform more general conclusions regarding other forms of gambling advertising.

### Study objective

The current study has the specific aim to evaluate whether opting out of receiving direct marketing messages reduces betting frequency, gambling expenditure and short‐term gambling harms. An EMA is a research paradigm well suited to this purpose. It collects data repeatedly over short time frames, in this case every other day over a 2‐week period, in a naturalistic setting. In the current study, this was characterised by querying regular sports bettors every 48 hours during a time when there were many possibilities for opportunistic bets on sports or races. EMAs have the advantage of minimising recall biases [33], having high ecological validity [34] and allowing an exploration of time‐course dynamics [35, 36]. EMAs have been used in gambling research previously: for example, to assess the effectiveness of app‐delivered craving management intervention [37] and in exploring how positive outcome expectancies contribute to gambling episodes [38].

In short, the present evidence suggests an association between direct marketing appeals and gambling behaviours that put people at risk of harm. However, causal evidence is lacking, as correlational data cannot definitively ascribe gambling choices and harm to the marketing appeals. This study explored a causal connection between direct marketing appeals and subsequent gambling behaviours and short‐term harms. Randomisation was used to allocate participants either to an intervention group that opted out of direct marketing or to a control group that continued receiving such communications.

## METHODS

Ethical approval for the overall EMA project, including the experimental component, was provided by CQUniversity Human Research Ethics Committee (approval no. 24276). De‐identified data will be made available upon reasonable request, subject to approval by Gambling Research Australia and the research ethics committee, and under a data‐use agreement. The analyses for the study were not pre‐registered and so should be considered exploratory.

### Participants

Australian residents from all states and territories were recruited via Qualtrics, a panel aggregator using independent market research panels. Participants were eligible if they: (i) were aged 18 years or older; (ii) resided in Australia; (iii) had an active online wagering account used within the previous 12 months; (iv) reported typically betting on sports or races at least once fortnightly in the past 12 months; (v) provided informed consent; (vi) agreed to complete seven EMA surveys over a 14‐day period; and (vii) indicated a willingness to opt out of direct marketing from their wagering operator(s) for the duration of the study.

Participants were excluded if they: (i) did not meet these criteria; (ii) failed the baseline attention‐check item; and/or (iii) did not complete at least one or more EMA survey.

**FIGURE 1:**
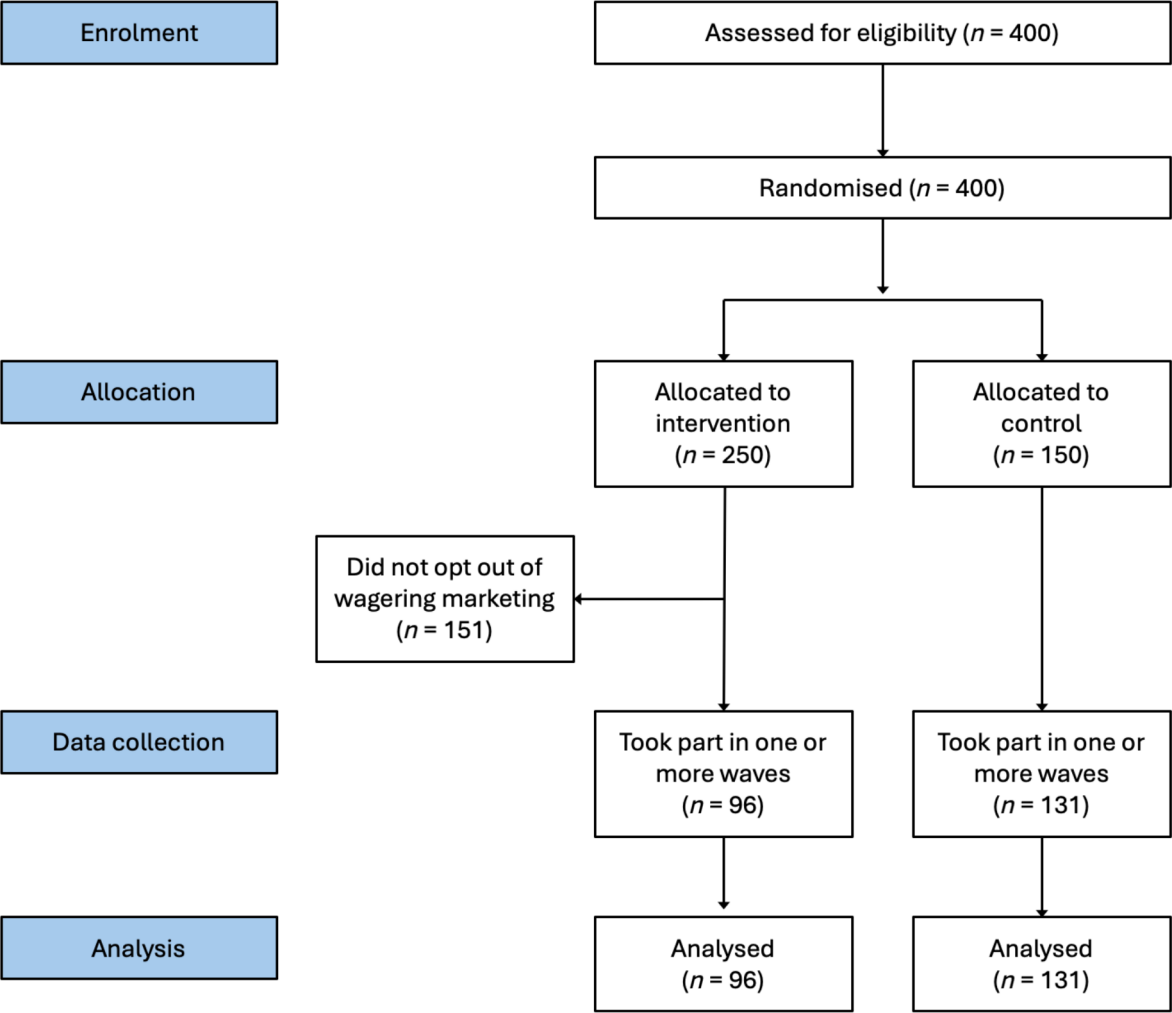
CONSORT flow diagram showing application of inclusion/exclusion criteria and participant progression.

The current experiment drew on a subset of participants who had previously completed a baseline survey, which was analysed separately for correlational purposes [14]. This experimental study was embedded within the same 14‐day EMA period as that of Hing *et al*. [14], but involved only the subset willing and eligible to opt out. The baseline included 1015 participants (58.8% men, 48.9% aged 18–39 years) who successfully completed this first survey, out of the 1189 people who started it. A quota was set for 400 people to take part in the experiment, and participants were invited to take part until the quota was full. A total of 615 participants were invited to take part in the experiment, and 405 (65.9%) opted to do so, slightly above the allowed quota. These 405 participants were selected at random and invited in batches until 400 had enrolled, consistent with the budget constraints of the study. Ultimately, 140 male and 87 female participants complied with the instructions and completed one or more EMA survey(s). Figure 1 shows the CONSORT (Consolidated Standards of Reporting Trials) flow diagram for the study.

### Design

This study was an exploratory naturalistic field experiment. The purpose was to investigate how exposure to wagering direct marketing appeals within a naturalistic setting affects betting behaviour and harm.

Participants were randomly assigned to: (i) the treatment group that opted out of direct marketing and provided proof of having done so (*n* = 96); or (ii) a control group of individuals who were willing to opt out of marketing but were not asked to do so (*n* = 131). Participants in the treatment group were requested to opt out of receiving emails, texts, app notifications, phone calls and any other direct marketing from all the Australian and overseas wagering operators with which they held an account. They were also requested to send proof of opting out from all operators. The proof was checked by a member of the research team and was received in the form of screenshots and emails forwarded by participants. The proof confirmed that the participant had opted out and generally included a message from the operator (e.g. ‘we're sorry to see you go’).

The remaining participants of the 400 invited were either assigned to the treatment group but were excluded from the study because they did not provide the required proof of having opted out (e.g. screenshots or confirmation emails) (*n* = 151) or did not take part in any of the EMA surveys (*n* = 22). Owing to the nature of the intervention (i.e. opting out of marketing), neither researchers nor participants could be blinded.

#### Randomisation procedures

Stratified randomisation was employed to enhance group comparability on key demographic and risk characteristics. Randomisation occurred using the randomiser function within the Qualtrics survey platform. The initial pool of eligible participants was stratified into cells based on three variables: (i) Problem Gambling Severity Index (PGSI) risk category (non‐/low‐risk gambling vs moderate/problem gambling); (ii) age group (18–35 vs 36+ years); and (iii) gender identity (male, female, other). Within each stratum, participants were randomly assigned to either the treatment group (60% of participants) or the control group (40% of participants).

All participants in the experiment, whether in the treatment or control groups, were given up to $50 in additional compensation prorated to the number of EMA surveys completed, in addition to the standard panel‐provider incentives they received for the baseline and EMA surveys (approx. $5 for each survey). This additional compensation was explained to participants during recruitment, when they were asked whether they were willing to opt out of direct marketing messages.

To assess potential selection bias in entry to the experiment, we compared the baseline characteristics of participants who were eligible in principle with those who were not. These comparisons showed minimal differences and are reported in Table S1.

### Materials and measures

The seven dependent‐variable assessments were administered using a 14‐day EMA design, with surveys completed every 48 hours.

#### Betting behaviour in the last 48 hours

In each of the seven EMA surveys, participants reported the number of bets they had placed in the previous 48 hours, and the total amount of money wagered on sports and race betting (AUD) during that period.

#### Short‐term betting harms in the last 48 hours

Short‐term harms were assessed using a 10‐item adapted version of the Short Gambling Harm Screen (SGHS) [39], modified to refer to harms experienced in the previous 48 hours and to specify harms occurring ‘due to betting’. Items were answered ‘yes’ or ‘no’ (e.g. ‘felt distressed about your betting’). The modified time‐frame SGHS demonstrated good internal consistency in the first EMA (Cronbach's *α* = 0.89). The scale also showed moderate convergent validity with the baseline PGSI scores (*r* = 0.582, *P* < 0.001, *n* = 186), which is somewhat lower than the approximate value of 0.75 reported in validation studies of the standard SGHS/GHS‐10 but still represents a substantial association.

The primary outcomes for the experimental analyses were the number of bets placed, expenditure and short‐term harms. The full EMA questionnaire, including all betting and harm items, is provided in Appendix S1.

### Procedure

The baseline survey that preceded the EMAs included questions on gambling behaviour, demographics and PGSI, and these answers were used for stratified random assignment to condition. After the baseline survey, both groups completed identical EMA surveys over a 2‐week period, which consisted of seven 15‐minute surveys completed every other day, covering 2 weeks. The survey links were delivered via email at 4 PM, and links remained valid until the next survey was opened 2 days later. All survey results were collected between Tuesday 18 July and Tuesday 29 August 2023. This period coincided with major Australian winter sporting events (Australian Football League, National Rugby League) and racing carnivals, to reflect high‐volume betting periods.

### Analytic approach

Analyses were conducted in R 4.0.2 [40] using linear mixed‐effects models with repeated EMA observations nested within participants. All data‐analysis code used for the statistical models and visualisations is provided in Appendix S2. The data for each wave were arranged into ‘long’ format, which was required for the linear mixed effects (LME) regression models that were used for analysis. Analyses were conducted using the *lme4* package [41] in R [40] with ‘UniqueID’ employed as a random factor to link participants across waves. This random factor was employed as a random intercept by participant, which controls for variance associated with individual differences. We attempted to employ random slopes to control for differential effect size over time, but this led to convergence issues with many models. Where models did converge, the fixed effects results were strongly similar for models that included the random slope compared with those that did not. Therefore, although some random‐slope specifications failed to converge, the random‐slope model that did converge is presented as model 3, for transparency.

As no pre‐registered statistical analysis plan or *a priori* sample size calculation was available, these analyses should be regarded as exploratory in nature.

To address zero‐inflation and positive skew in the statistical models, we applied Yeo–Johnson transformations (*λ* = −1 for number of bets; *λ* = −0.1 for expenditure and harms). Unlike Box–Cox transformation, this method accommodates zero‐values and was selected via standard diagnostic checks to ensure residual normality. For visualisation purposes only, we report trimmed means (excluding the upper and lower 10% of the distribution) to pevent extreme outliers from obscuring group‐level trends (raw distributions with individual data points are shown in Supplementary Figures [Link], [Link] and S3). All survey items required a response, resulting in no missing data. We examined intervals between successive EMA submissions to check for surveys completed too close together. Across 2836 observations, the mean interval was 1.98 days (median = 2 days), and only 8.6% occurred within 24 hours of the prior EMA. These short gaps reflect end‐of‐window and start‐of‐window completions within the 48‐hour survey periods, rather than duplicate reporting, so no data were removed.

We also constructed two exploratory indicators: one comparing planned spend in wave *t* with actual spend in wave *t* + 1 (‘excess spend’), and one capturing the proportion of spend participants reported placing ‘on impulse’. These measures were expected to align and to correlate with PGSI; however, they showed poor reliability and weak associations and were therefore not used in the primary analyses.

Baseline characteristics for the treatment and control groups were compared descriptively (means and proportions) to assess balance after randomisation, and consistent with CONSORT recommendations, no significance testing was performed for baseline differences.

#### Model specification

For each of the three dependent variables, we report three models:

model 1, a main effect for experimental condition and a random intercept for participants;
model 2, model 1 plus a main effect and interaction for wave;
model 3, model 2 plus a random slope for wave that could be correlated with the random intercept.

Thus, the first model fits only a mean difference between experimental and control conditions and mean individual differences; the second model additionally allows for a linear time effect that could be different for the two groups; and the third model additionally allows for random variability in the gradient of the time effect across participants. Note that wave is classified as a continuous variable. *P*‐values reported in the text are drawn from the first model for each dependent variable, noting that the *P*‐values are generally also similar in the second and third models.

## RESULTS

### Sample characteristics

Table 1 shows the demographic and PGSI categories by condition. As expected by random assignment, baseline demographic characteristics were similar across conditions.

**TABLE 1:** Demographics and gambling characteristics by condition at baseline.

Variable	Level	Control group (*n* = 131)	Treatment group (*n* = 96)	Inferential statistic
Gender, *n* (%)	Men	79 (60.3)	61 (63.5)	χ^2^(1) = 0.128, *P* = 0.721
	Women	52 (39.7)	35 (36.5)	
Age, years, mean (SD)		45.2 (15.4)	45.6 (15.0)	*t*(208) = 0.866, *P* = 0.388
Main language spoken at home, *n* (%)	English	128 (97.7)	94 (97.9)	χ^2^(1) = 0, *P* = 1.0
	Another language	3 (2.3)	2 (2.1)	
PGSI, *n* (%)	No problem	33 (25.2)	25 (26.8)	χ^2^(3) = 0.82, *P* = 0.845
	Low risk	27 (20.6)	24 (25.0)	
	Moderate risk	39 (29.8)	25 (26.0)	
	Problem	32 (24.4)	22 (22.9)	
	Moderate risk or problem	71 (54.2)	47 (49.0)	χ^2^(1) = 0.42, *P* = 0.518
	No problem or low risk	60 (45.8)	49 (51.0)	
Race betting frequency, *n* (%)	Weekly or more	92 (70.2)	57 (59.4)	χ^2^(1) = 2.43, *P* = 0.119
	Less than weekly	39 (29.8)	39 (40.6)	
Sports betting frequency, *n* (%)	Weekly or more	77 (58.8)	65 (67.7)	χ^2^(1) = 1.52, *P* = 0.217
	Less than weekly	54 (41.2)	31 (32.3)	

*Note*: Chi‐square includes Yates’ continuity correction, hence there is a chi‐square value of 0 and a *P*‐value for 1 for main language spoken at home despite the percentages not being perfectly equal.

Abbreviation: PGSI = Problem Gambling Severity Index.

Completion rates of the seven EMA surveys were high in both conditions (Table 2). Among participants who completed at least one EMA (*n* = 131 control, *n* = 96 treatment), the number of completed surveys per wave ranged from 93 to 103 (71%–79%) in the control group and from 79 to 86 (82%–90%) in the treatment group. Completion numbers were relatively stable across the 14‐day period, with no indication of progressive dropout in either condition.

**TABLE 2:** Number of participants per wave per condition.

Wave	Date (all in 2023)	Control group	Treatment group
Baseline	Tues 18th July to Sat 5th August	131	96
1	Tues 15th August to Thurs 17th August	102	83
2	Thurs 17th August to Sat 19th August	93	79
3	Sat 19th August to Mon 21st August	94	81
4	Mon 21st August to Wed 23rd August	100	81
5	Wed 23rd August to Fri 25th August	99	86
6	Fri 25th August to Sun 27th August	97	79
7	Sun 27th August to Tue 29th August	103	85

*Note*: Participants who agreed to take part in the ecological momentary assessment (EMA) but did not complete any EMA studies are not included in the baseline figure.

### Descriptive statistics

#### Number of bets

**FIGURE 2:**
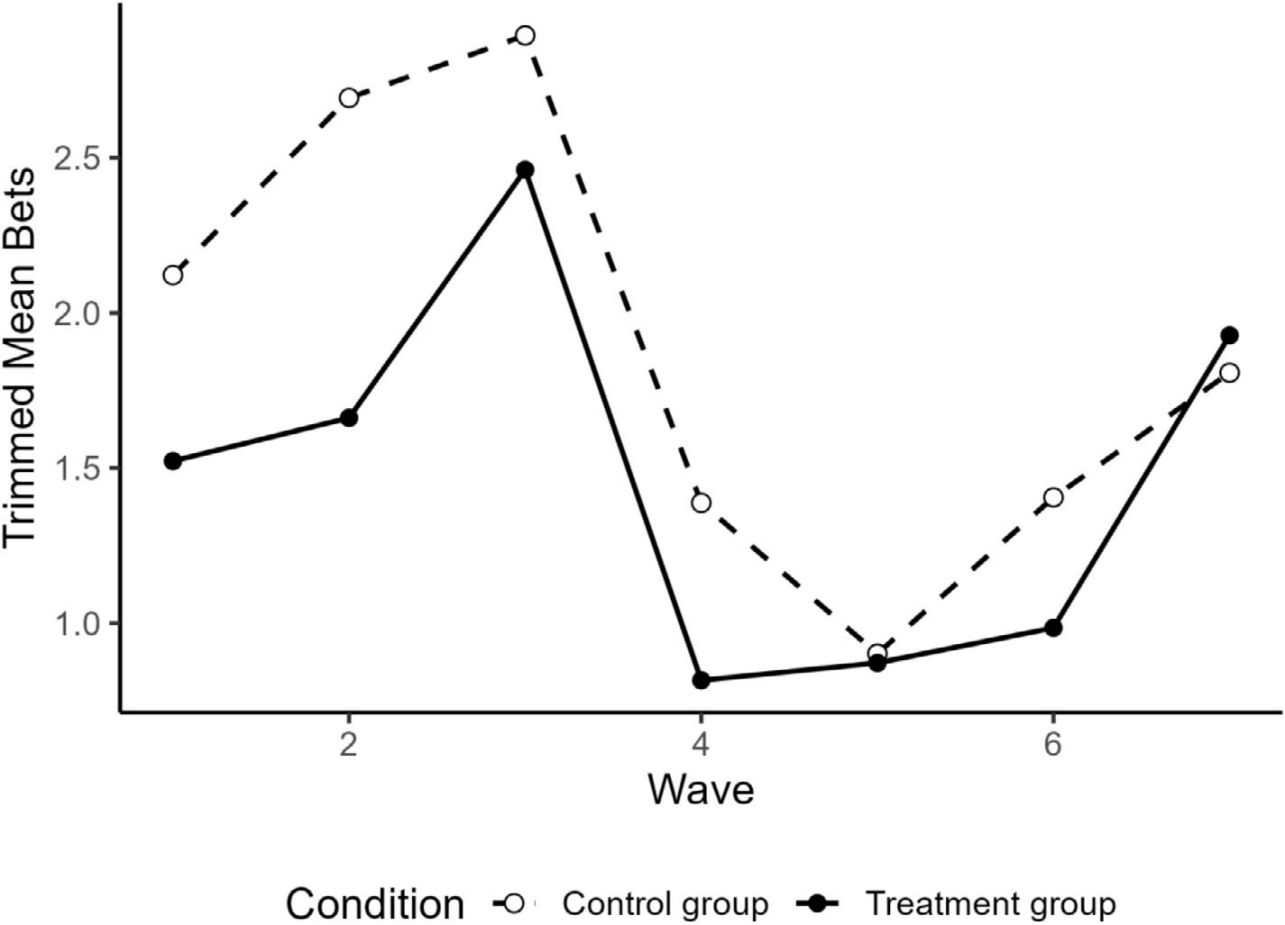
Mean number of bets for the control and treatment groups over time.

The mean number of bets throughout the study was 12.5 bets for the control condition (1.78 per wave) and 9.6 bets for the treatment condition (1.37 per wave). That is, on average, those who opted out of direct messages placed 23% fewer bets than those who had not opted out. Figure 2 shows the average number of bets by condition by wave, with the highest points associated with weekends (waves 2, 3, 6 and 7).

#### Spend

**FIGURE 3:**
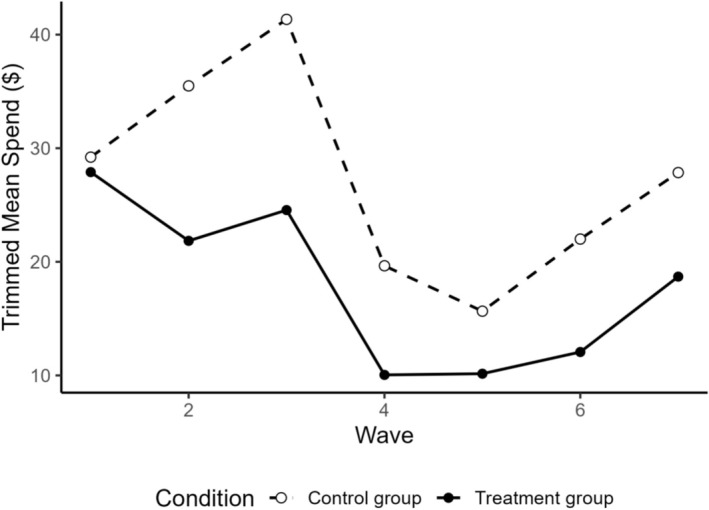
Mean spend for the control and treatment groups over time.

The mean per‐participant spend over the course of the seven waves was $185.50 for the control group ($26.50 per wave) and $113.40 for the treatment group ($16.20 per wave), with peaks on the weekends (waves 2, 3, 6 and 7). On average, those who had opted out of direct messages spent 39% less than those in the control group (Figure 3).

#### Harms

**FIGURE 4:**
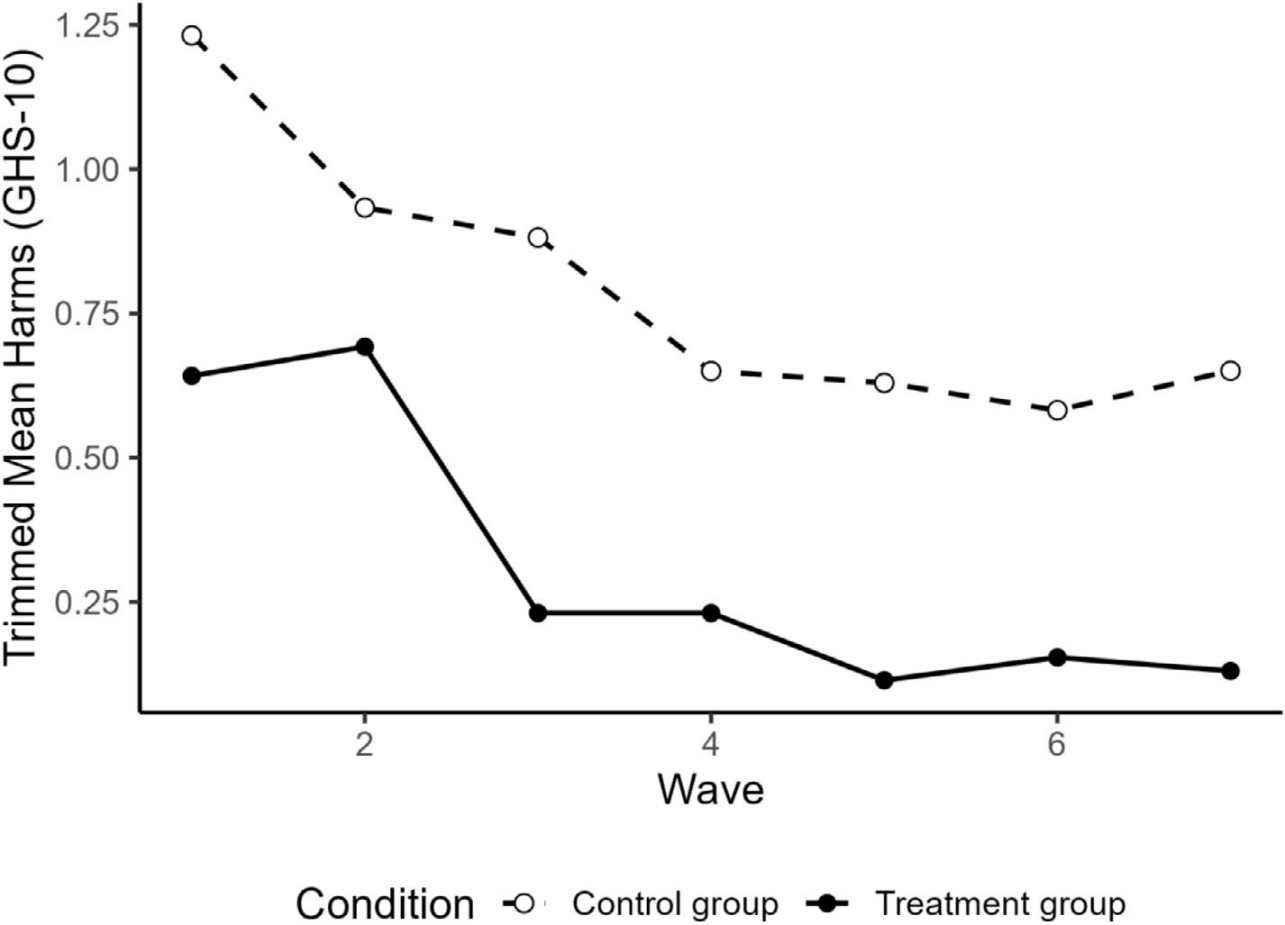
Mean number of harms for the control and treatment groups over time.

The mean number of short‐term harms reported per wave in the control condition was 0.77, compared with 0.25 per wave for the treatment condition. This represents a decrease of 67% in short‐term harm for those who opted out of receiving direct messages (Figure 4).

### Models

Tables 3, 4, 5 summarise the LME models predicting the number of bets, spend and short‐term harm. For transparency, we report three specifications for each outcome: model 1 (main effect of condition); model 2 (condition × wave interaction); and model 3 (random slope for wave).

**TABLE 3:** Models investigating the effect of experimental intervention on number of bets (Yeo–Johnson transformed with lambda = −1).

Predictors	Model 1			Model 2			Model 3		
	*B*	95% CI	*P*	*B*	CI	*P*	*B*	95% CI	*P*
(Intercept)	0.56	0.50, 0.62	**<0.001**	0.67	0.59, 0.75	**<0.001**	0.67	0.59, 0.76	**<0.001**
Exp. (test)	−0.11	−0.20, −0.03	**0.011**	−0.20	−0.31, −0.08	**0.001**	−0.19	−0.32, −0.07	**0.002**
Wave				−0.03	−0.04, −0.02	**<0.001**	−0.03	−0.04, −0.01	**<0.001**
Exp. (test) × wave				0.02	0.00, 0.04	**0.035**	0.02	−0.00, 0.04	0.060
**Random effects**									
*σ* ^2^	0.12			0.12			0.11		
*τ*_00_	0.09_UniqueID_			0.09_UniqueID_			0.11_UniqueID_		
*τ*_11_							0.00_UniqueID.Wave_		
*ρ*_01_							−0.48_UniqueID_		
ICC	0.42			0.43			0.46		
*n*	227_UniqueID_			227_UniqueID_			227_UniqueID_		
Obs.	1282			1282			1282		
Marg. *R* ^2^/Cond. *R* ^2^	0.016/0.433			0.024/0.440			0.024/0.468		

*Note*: Coefficients are unstandardised, but the dependent variable is transformed so coefficients cannot be interpreted as raw differences in terms of number of bets. Random effects: *σ* ^2^ = residual variance; *τ*_00_ = random intercept variance (participant); *τ*_11_ = random slope variance for wave (participant; Model 3 only); *ρ*_01_ = intercept‐slope correlation (Model 3 only); ICC = proportion of total variance attributable to between‐person differences. Bold values indicate *p* < 0.05 (two‐tailed).

**TABLE 4:** Models investigating the effect of experimental intervention on spend (Yeo–Johnson transformed with lambda = −0.1).

Predictors	Model 1			Model 2			Model 3		
	*B*	95% CI	*P*	Predictors	*B*	95% CI	*P*	Predictors	*B*
(Intercept)	2.02	1.81, 2.22	**<0.001**	2.38	2.11, 2.65	**<0.001**	2.38	2.10, 2.66	**<0.001**
Exp. (test)	−0.53	−0.84, −0.21	**0.001**	−0.74	−1.15, −0.33	**<0.001**	−0.73	−1.16, −0.31	**0.001**
Wave				−0.09	−0.14, −0.05	**<0.001**	−0.09	−0.14, −0.04	**<0.001**
Exp. (test) × wave				0.05	−0.01, 0.12	0.109	0.05	−0.02, 0.12	0.151
**Random effects**									
*σ* ^2^	1.40			1.38			1.32		
*τ*_00_	1.11_UniqueID_			1.10_UniqueID_			1.29_UniqueID_		
*τ*_11_							0.01_UniqueID.Wave_		
*ρ*_01_							−0.38_UniqueID_		
ICC	0.44			0.45			0.47		
*n*	227_UniqueID_			227_UniqueID_			227_UniqueID_		
Obs.	1262			1262			1262		
Marg. *R* ^2^/Cond. *R* ^2^	0.027/0.458			0.035/0.465			0.035/0.487		

*Note*: Coefficients are unstandardised, but the dependent variable is transformed so coefficients cannot be interpreted as raw differences in terms of amount spent. Random effects: *σ* ^2^ = residual variance; *τ*_00_ = random intercept variance (participant); *τ*_11_ = random slope variance for wave (participant; Model 3 only); *ρ*_01_ = intercept‐slope correlation (Model 3 only); ICC = proportion of total variance attributable to between‐person differences. Bold values indicate *p* < 0.05 (two‐tailed).

**TABLE 5:** Models investigating the effect of experimental intervention on harm (Yeo‐Johnson transformed with lambda = −0.1).

Predictors	Model 1			Model 2			Model 3		
	*B*	95% CI	*P*	Predictors	*B*	95% CI	*P*	Predictors	*B*
(Intercept)	0.50	0.41, 0.60	**<0.001**	0.64	0.53, 0.75	**<0.001**	0.65	0.52, 0.77	**<0.001**
Exp. (test)	−0.22	−0.36, −0.07	**0.004**	−0.23	−0.40, −0.06	**0.007**	−0.24	−0.43, −0.04	**0.017**
Wave				−0.03	−0.05, −0.02	**<0.001**	−0.04	−0.05, −0.02	**<0.001**
Exp. (test) × wave				0.00	−0.02, 0.03	0.699	0.01	‐0.02, 0.03	0.701
**Random effects**									
*σ* ^2^	0.15			0.15			0.13		
*τ*_00_	0.27_UniqueID_			0.27_UniqueID_			0.40_UniqueID_		
*τ*_11_							0.00_UniqueID.Wave_		
*ρ*_01_							−0.61_UniqueID_		
ICC	0.64			0.65			0.69		
*n*	227_UniqueID_			227_UniqueID_			227_UniqueID_		
Obs.	1262			1262			1262		
Marg. *R* ^2^/Cond. *R* ^2^	0.027/0.652			0.036/0.662			0.037/0.700		

*Note*: Coefficients are unstandardised, but the dependent variable is transformed so coefficients cannot be interpreted as raw differences in terms of harm scores. Random effects: *σ* ^2^ = residual variance; *τ*_00_ = random intercept variance (participant); *τ*_11_ = random slope variance for wave (participant; Model 3 only); *ρ*_01_ = intercept‐slope correlation (Model 3 only); ICC = proportion of total variance attributable to between‐person differences. Bold values indicate *p* < 0.05 (two‐tailed).

Across all outcomes, the opt‐out intervention was associated with significantly lower gambling intensity. In the primary specification (model 1), those who opted out of receiving direct messages placed significantly fewer bets (*b* = −0.11; 95% CI = −0.20, −0.03; *P* = 0.011), bet a significantly lower amount of money (*b* = −0.53; 95% CI = −0.84, −0.21; *P* = 0.001) and showed lower levels of short‐term harms (*b* = −0.22; 95% CI = −0.36, −0.07; *P* = 0.004).

When including the time effect and interaction (model 2), results varied by outcome. For betting frequency, a small but significant condition × wave interaction was observed (*b* = 0.02; 95% CI = 0.00, 0.04; *P* = 0.035), suggesting that the between‐group difference in betting frequency attenuated over the 14 days, with the largest differences observed in the early waves. However, no significant interactions were found for expenditure (*P* = 0.109) or short‐term harms (*P* = 0.699), suggesting that the reduction in spend and harm for the opt‐out group remained consistent across the study period. Across outcomes, adding a main effect of wave and a condition × wave interaction (model 2), and then allowing random slopes over the wave (model 3), left the estimated main effect of condition essentially unchanged; the point estimates and 95% confidence intervals for the condition effect in models 2 and 3 were very similar to those from the primary model (Tables 3, 4, 5).

## DISCUSSION

This naturalistic field experiment employed an EMA design to survey sports and race bettors every other day over a period of 2 weeks, coinciding with numerous betting events. By random assignment, some of the participants were asked to opt out of receiving direct marketing appeals and provide proof of having done so (e.g. via email confirmations or screenshots). Our purpose was to see whether opting out of wagering direct marketing had effects on betting behaviours and the short‐term betting‐related harms experienced. One potential argument for direct marketing is that it reflects competition for customers between wagering operators and thus creates value for consumers. However, evidence of more intensive gambling and greater harm in the presence of such marketing undermines this narrative.

The principal result of the present study is that people who had opted out of direct marketing placed 23% fewer bets, had 39% lower spending and reported 67% fewer short‐term harms than people in the control group who continued to receive such communications. This consistency across measures is a substantial indication that direct marketing increases the intensity of betting, spending and harm among existing customers. Wagering operators commonly justify direct marketing as ordinary competitive behaviour aimed at ‘winning customers’, but our findings instead show that direct marketing amplifies betting and harm within a customer base that operators already have. Moreover, the size of the effects suggests that direct marketing is likely to be a highly cost‐effective means of extracting more spend from gambling customers and that this spending results in additional harm. Baseline data and prior work on this cohort suggest that sports and race betting typically occur alongside other gambling products, such as lotteries, electronic gambling machines (EGMs) or other online gambling, so the EMA outcomes should be interpreted as changes in one important component of a broader gambling portfolio, rather than representing the entire gambling activity of the participants.

Across the study period, the trajectories for both groups exhibited the same temporal pattern, with clear peaks during weekends and major sporting fixtures (Figures 2, 3, 4). This indicates that the intervention did not fundamentally alter when people chose to bet. Instead, opting out of direct marketing consistently reduced the intensity of betting, spending and harms at each of these natural high‐opportunity points. In other words, direct marketing appears to amplify existing betting rhythms rather than create new ones, and removing it lowers the amplitude of these fluctuations without changing their form. Relatedly, there was no *a priori* reason to expect progressively larger effects across EMA waves, as removing situational cues and incentives would most plausibly reduce betting intensity immediately, rather than through a gradual withdrawal process.

### Causal role

This study is important in providing evidence of a causal role for direct marketing in gambling intensification and consequent harm. Most past research has provided correlation evidence, that while compelling, can potentially be explained by factors such as reverse causation and third variables [3, 6, 12, 15]. For example, gambling operators may provide direct marketing messages more frequently to people who spend more and experience more harm. Moreover, people who are losing money may seek out direct marketing opportunities and be more likely to remember them when asked.

The current study has the advantage of demonstrating causal inference, as exposure to direct marketing was manipulated experimentally via random assignment. With the limitations and caveats noted below, the only difference between conditions is the context of this random assignment, and not pre‐existing factors such as the actions of operators or individual factors such as the number of betting accounts people have or their impulsive personal tendencies.

The current study also has some advantages over prior approaches owing to biases inherent in retrospective self‐reporting. People are more likely to have bias in recall when considering more distant past events (e.g. in the last 12 months) [36] and show social desirability in responding [33], such as minimising losses or difficulties. The approximate 48‐hour time frame of recall for the EMA is more likely to minimise such biases.

### Policy and regulation

Policy options exist that can reduce exposure while preserving consumer choice. Proportionate measures could include: (i) an opt‐in requirement for direct marketing (with simple, repeated opportunities to opt out); (ii) caps on message frequency; and (iii) limits on some inducements or urgency/social‐pressure content within direct messages (for example, bonus‐bet framing and ‘limited time’ prompts). Such measures recognise that some consumers may engage with promotional offers in a deliberate, value‐seeking way, while reducing the volume and intensity of cues that may escalate betting for others.

### Limitations

It is notable that in the experiment, some of the participants in the test condition likely still received some marketing, including potentially direct marketing from wagering affiliates who were promoting the operator's inducements. Although these participants were instructed to opt out from all wagering operators and were required to provide evidence of doing so, some residual exposure to direct marketing is still possible; for example, if participants overlooked an account, continued to receive affiliate promotions, or encountered marketing through social media or general digital advertising. Importantly, the experimental contrast was designed to represent a substantial reduction in direct marketing rather than complete elimination, and any remaining exposure would be expected to attenuate rather than inflate the observed intervention effects. Consequently, this study is not a demonstration of ‘no’ versus ‘some’ direct marketing, but rather ‘some’ versus ‘more’ direct marketing.

Some additional important limitations are evident in interpreting the results. First, only people who were willing to opt out of receiving messages were eligible for inclusion in the experiment. Restrictions on direct marketing because of policy action would apply to all persons, and not just those who are willing to opt out. The EMA surveys captured only sports and race betting. We did not measure expenditure on other gambling products (e.g. EGMs, casino gambling, lotteries), so it is possible that some gambling activity or spending was displaced to other forms during the study period. The spend measure reflected the total amount that participants reported placing on bets, which may have included some bonus or promotional credits, although any such inclusion would be expected to dilute rather than inflate the observed difference between the conditions. Further, the study had a short duration of 2 weeks, and thus it is not clear whether behaviour would be persistently changed with a long‐term reduction in direct marketing. People who took part in the study were necessarily aware that we were asking about their betting, and this could have had some effect on their behaviour. Of course, we did not tell people in advance that they were part of an experimental study, nor that people would be participating in the study under different conditions. Nevertheless, the participants’ awareness of the study may have changed their behaviour owing to social desirability concerns or other factors. For example, participants in the opt‐out condition may have reduced their betting because study participation prompted the idea of taking a temporary break, rather than because the absence of direct marketing reduced their gambling intensity. Furthermore, while attrition was very low, there is always some risk of findings being skewed at least somewhat by people who skip EMA surveys.

Although stratified randomisation was employed, substantial post‐randomisation attrition, particularly among participants allocated to the treatment group who did not provide proof of opting out, may have led to minor imbalances between the arms (for details, see Table 1), and this should be considered when interpreting the findings. Despite these shortcomings, the study had the strength of being, to our understanding, the first experimental field study of the effects of direct marketing messages on betting behaviour and harmful outcomes. It has high ecological validity through being situated in a real‐world environment, with regular bettors placing bets during a period with many relevant sporting and racing events. Other exploratory derived variables (e.g. planned vs actual spend, proportion of spend placed on impulse) were examined but showed poor reliability and were therefore not included in the primary analyses.

### Future directions

The findings add to a growing literature suggesting that direct marketing may contribute to intensified betting and harm. An ideal econometric study might examine changes in policy in one jurisdiction against similar jurisdictions that have not banned or limited direct marketing [32]. Other valuable research might examine before versus after effects (i.e. interrupted time series) of partial or total bans on direct marketing to see if it affects long‐term behaviours and harm [28]. Although the present study focused on sports and race betting, it is plausible that similar amplification effects of direct marketing could occur in other online gambling products, and future research could test this using comparable naturalistic or experimental field designs.

## CONCLUSION

Experimental evidence gathered in the current study suggests that direct marketing for wagering is not just a vehicle for brands to gain market share. Instead, or in addition, direct marketing acts on gamblers to increase the number of bets they make, increase their spending and add to the harm that they experience. This research contributes to an existing correlational evidence base that also suggests direct marketing has these deleterious outcomes.

## AUTHOR CONTRIBUTIONS

**Matthew Rockloff:** Conceptualization (lead); funding acquisition (equal); methodology (equal); writing—original draft (lead); writing—review and editing (equal). **Matthew Browne:** Conceptualization (equal); data curation (lead); formal analysis (lead); methodology (equal); software (equal); visualization (equal); writing—original draft (equal); writing—review and editing (equal). **Nerilee Hing:** Conceptualization (equal); funding acquisition (lead); investigation (equal); methodology (equal); project administration (equal); resources (equal); supervision (equal); writing—review and editing (equal). **Alex M. T. Russell:** Conceptualization (equal); data curation (equal); investigation (equal); methodology (lead); writing—original draft (equal); writing—review and editing (equal). **Vijay Rawat:** Funding acquisition (equal); methodology (equal); project administration (equal); resources (equal); software (equal); writing—review and editing (equal). **Philip Newall:** Writing—original draft (equal); writing—review and editing (equal).

## DECLARATION OF INTERESTS

M.R. has received research funds from Gambling Research Australia, the Victorian Responsible Gambling Foundation, the ACT Gambling and Racing Commission, the Queensland Treasury, the Victorian Treasury, the NSW Responsible Gambling Fund, the NSW Office of Liquor & Gaming, the Tasmanian Department of Treasury and Finance, the SA Department of Human Services, the New Zealand Ministry of Health, the Department of Families, Housing, Community Services and Indigenous Affairs, the Alberta Gambling Research Institute and the First Nations Foundation. He declares no conflicts of interest in relation to this article. M.B. has received research funds from Gambling Research Australia, the Victorian Responsible Gambling Foundation, the Queensland Government Department of Health, the South Australian Government, the Australian Department of Social Services, and the New Zealand Ministry of Health. He declares no conflicts of interest in relation to this article. N.H. has received funding in the last 5 years from Gambling Research Australia, the Victorian Responsible Gambling Foundation, the Victorian Department of Justice, the NSW Responsible Gambling Fund and NSW Office of Responsible Gambling, the New Zealand Ministry of Health, the Queensland Department of Justice and Attorney‐General, the South Australian Independent Gambling Authority, the South Australian Government, the ACT Gaming and Racing Commission, the Australian Research Council, Australia's National Research Organisation for Women's Safety. She declares that she has no conflicts of interest in relation to this article. A.R. has received funding from the Victorian Responsible Gambling Foundation, the Victorian Department of Justice and Community, the New South Wales Office of Responsible Gambling, the ACT Gambling and Racing Commission, the South Australian Government, the Queensland Department of Justice, Gambling Research Australia, the New Zealand Ministry of Health, the Australian Communications and Media Authority and the Alberta Gambling Research Institute. He has had travel expenses paid to present research by the Victorian Responsible Gambling Foundation, PsychMed, the ACT Gambling and Racing Commission and the Hawthorn Hawks Football Club Players Association. He has received an honorarium from Movember for assessing applications for funding and consulting fees from the Victorian Responsible Gambling Foundation. He declares no conflicts of interest in relation to this article. V.R. has received research funding from Gambling Research Australia, the New South Wales Responsible Gambling Fund and the Victorian Responsible Gambling Foundation. He declares no conflicts of interest in relation to this article. P.N. was a member of the Advisory Board for Safer Gambling from 2021 to 2025—an advisory group of the Gambling Commission in Great Britain. In the last 3 years, P.N. has been a named researcher on projects funded by the Academic Forum for the Study of Gambling, Alberta Gambling Research Institute, BA/Leverhulme, Canadian Institute for Health Research, Clean Up Gambling, Gambling Research Australia and the Victorian Responsible Gambling Foundation. P.N. has received honoraria for reviewing from the Academic Forum for the Study of Gambling and the Belgium Ministry of Justice, travel and accommodation funding from the Alberta Gambling Research Institute and the Economic and Social Research Institute, and open‐access fee funding from the Academic Forum for the Study of Gambling and Greo Evidence Insights. He declares no conflicts of interest in relation to this article.

## Supporting information


**Appendix S1.**Short EMA survey on direct and affiliate wagering marketing.

**Appendix S2.** EMA analysis code (R 4.0.2).

**Figure S1.** Raw bets.

**Figure S2.** Raw spend.

**Figure S3.** Raw harms.

**Table S1.** Baseline characteristics by eligibility in principle for the experiment (participants with complete data, N = 1,015).

## Data Availability

De‐identified data will be made available upon reasonable request, subject to approval by Gambling Research Australia and the research ethics committee, and under a data‐use agreement.
