# Supplementary material for: Direct gambling marketing, direct harm: A randomised experiment
Source: Addiction. 2026 Mar 18;121(7):1907–19. doi: 10.1111/add.70369 (PMC13291081; doi:10.1111/add.70369)
Supplement: Supplementary file 1 — Appendix S1.Short EMA survey on direct and affiliate wagering marketing. [file ADD-121-1907-s006.docx]

**Direct and Affiliate Wagering Marketing**

**Short EMA Survey**

Thank you for previously completing the first (baseline) survey for this study. This short survey will take you about 5 minutes to complete. **Please look out for additional short surveys that will be sent to you every 48 hours.**

Remember – to maximise the value of the gift vouchers you will receive, you need to complete the short surveys and forward us any emails, text messages or notifications that you received from wagering operators, tipsters and other betting-related services in the last 48 hours.

The last page of the survey has these instructions. We want to make sure you're rewarded for your participation.

E1. **In the last 48 hours**, how many **bets** did you place on sports and races?

(text box) bets

E2. **In the last 48 hours**, about how much **money** did you **place** on sports and race bets?

**Enter $**

(ASK all respondents who have bet in the last 48 hours, i.e. entered anything other than “0” at E1)

E3. **In the last 48 hours**, about what **percentage** of your sports bets would you describe as:

1. Planned in advance ___ %
2. Placed on impulse ___ %

Total 100%

*(Enter amounts to total 100%)*

E4. **In the last 48 hours**, how many of each of the following **wagering inducements** did you take up?

**If none, please enter 0.**

|  | Enter number |
| --- | --- |
| Sign-up offer (to open a betting account) |  |
| Refer-a-friend offer |  |
| Match (or partially match) your stake/deposit (with bonus bets) |  |
| Multi-bet offer (bonus bet, refund or cash if multi bet fails by one leg) |  |
| Bonus or better odds/winnings (e.g., odds boosts) |  |
| Refund/stake-back offer (some money back if bet doesn’t win) |  |
| Cash-out early on a multi-bet |  |
| Happy hour with better odds or winnings |  |
| Rewards program run by wagering operator |  |
| Other (please specify) |  |

The next few questions are about **wagering operators** who provide sports betting and race betting services to customers (e.g., Sportsbet, Bet365, Ladbrokes, etc).

EWO1. **In the last 48 hours**, how often did you see **advertisements or promotions** for **wagering** **operators** in the following media?

|  | Never | A few times | Often |
| --- | --- | --- | --- |
| Social media (e.g., Twitter, Facebook, TikTok, etc) |  |  |  |
| Streaming services used by online influencers (e.g., Twitch, YouTube) |  |  |  |
| Other online media (e.g., websites, online adverts, etc) |  |  |  |
| Traditional forms of media (e.g., TV and paid content streaming like Kayo, radio, print, billboard, cinema etc) |  |  |  |

EWO2. **In the last 48 hours**, about how many **emails, text messages, notifications and phone calls** did you receive from the **wagering operators you have an account with.** These include (pipe through names of wagering operators from WO1)?

**If none, please enter 0.**

|  | Enter number |
| --- | --- |
| 1. Emails |  |
| 1. Text messages |  |
| 1. App notifications |  |
| 1. Phone calls |  |

(ASK ALL UNLESS “0” TO ALL ITEMS IN EWO2)

EWO3. **In the last 48 hours**, about how many of these messages (emails, text messages, notifications and phone calls) that you received from **wagering operators** had the following features? These wagering operators include (pipe through names of wagering operators from WO1).

|  | None of them | Some of them | Most of them |
| --- | --- | --- | --- |
| The messages were relevant to **sports or sports betting** |  |  |  |
| The messages were relevant to **racing or race betting** |  |  |  |
| The messages promoted an **inducement to bet**, e.g., offers with bonus bets, money-back offers, improved odds, cash rebates, or rewards points |  |  |  |
| The messages were **customised** i.e., based on personalised knowledge about you or your betting |  |  |  |

(ASK ALL UNLESS THEY REPORTED NO BETS AT E1)

EWO4. **In the last 48 hours**, how much was your betting **influenced** in the following ways by tips, information or messages from the **wagering operators** you use (pipe through names of paid tipsters from WO1)?

|  | Not at all | A little | A lot |
| --- | --- | --- | --- |
| How much I bet |  |  |  |
| My betting selections |  |  |  |
| The inducements I used |  |  |  |
| Which operators I bet with |  |  |  |

Our next few questions are about **paid tipsters**. Paid tipsters are services including tipsters and tipping websites where you **pay a subscription fee** to receive tips and other information to inform your betting.

EPT1. **In the last 48 hours**, how often did you see **advertisements or promotions** for **paid tipsters** in the following media?

|  | Never | A few times | Often |
| --- | --- | --- | --- |
| Social media (e.g., Twitter, Facebook, TikTok, etc) |  |  |  |
| Streaming services used by online influencers (e.g., Twitch, YouTube) |  |  |  |
| Other online media (e.g., websites, online adverts, etc) |  |  |  |
| Traditional forms of media (e.g., TV and paid content streaming like Kayo, radio, print, billboard, cinema etc) |  |  |  |

(ASK ALL UNLESS THEY REPORTED NO PAID TIPSTER SERVICES AT PT1a)

EPT2. **In the last 48 hours**, about how many **emails, text messages, notifications and phone calls** did you receive from the **paid tipsters you use.** These include (pipe through names of paid tipsters from PT1b)?

**If none, please enter 0.**

|  | Enter number |
| --- | --- |
| 1. Emails |  |
| 1. Text messages |  |
| 1. App notifications |  |
| 1. Phone calls |  |

(ASK ALL UNLESS THEY REPORTED “0” TO ALL ITEMS IN EPT2)

EPT3. **In the last 48 hours**, about how many of these messages (emails, text messages, app notifications and phone calls) that you received from **paid tipsters** had the following features? These paid tipsters include (pipe through names of paid tipsters from PT1b).

|  | None of them | Some of them | Most of them |
| --- | --- | --- | --- |
| The messages were relevant to **sports or sports betting** |  |  |  |
| The messages were relevant to **racing or race betting** |  |  |  |
| The messages promoted an **inducement to bet**, e.g., offers with bonus bets, money-back offers, improved odds, cash rebates, or rewards points |  |  |  |
| The messages were **customised** i.e., based on personalised knowledge about you or your betting |  |  |  |

(ASK ALL UNLESS THEY REPORTED NO BETS AT E1 AND NO PAID TIPSTER SERVICES AT PT1a)

EPT4. **In the last 48 hours**, how much was your betting **influenced** in the following ways by tips, information or messages from the **paid tipsters** you use (pipe through names of paid tipsters from PT1b)?

|  | Not at all | A little | A lot |
| --- | --- | --- | --- |
| How much I bet |  |  |  |
| My betting selections |  |  |  |
| The inducements I used |  |  |  |
| Which wagering operators I bet with |  |  |  |

Our next few questions are about **free betting info services**. These include services, such as affiliate marketers, tipsters, betting communities, odds comparison sites, expert review sites, and sports/racing news sites, that **do not charge a fee** for betting info. They do **NOT** include paid tipsters who charge a subscription fee to provide tips and betting news.

EFAS1 **In the last 48 hours**, how often did you see **advertisements or promotions** for **free betting info services** in the following media?

|  | Never | A few times | Often |
| --- | --- | --- | --- |
| Social media (e.g., Twitter, Facebook, TikTok, etc) |  |  |  |
| Streaming services used by online influencers (e.g., Twitch, YouTube) |  |  |  |
| Other online media (e.g., websites, online adverts, etc) |  |  |  |
| Traditional forms of media (e.g., TV and paid content streaming like Kayo, radio, print, billboard, cinema etc) |  |  |  |

(ASK ALL UNLESS THEY REPORTED NO FREE BETTING INFO SERVICES AT FAS1a)

EFAS2. **In the last 48 hours**, about how many **emails, text messages, notifications and phone calls** did you receive from the **free betting info services you use.** These include (pipe through names of free services from FAS1b)?

**If none, please enter 0.**

|  | Enter number |
| --- | --- |
| 1. Emails |  |
| 1. Text messages |  |
| 1. App notifications |  |
| 1. Phone calls |  |

(ASK ALL UNLESS THEY REPORTED “0” TO ALL ITEMS IN EFAS2)

EFAS3. **In the last 48 hours**, about how many of these messages (emails, text messages, notifications and phone calls) that you received from **free betting info services** had the following features? These services include (pipe through names of free services from FAS1b).

|  | None of them | Some of them | Most of them |
| --- | --- | --- | --- |
| The messages were relevant to **sports or sports betting** |  |  |  |
| The messages were relevant to **racing or race betting** |  |  |  |
| The messages promoted an **inducement to bet**, e.g., offers with bonus bets, money-back offers, improved odds, cash rebates, or rewards points |  |  |  |
| The messages were **customised** i.e., based on personalised knowledge about you or your betting |  |  |  |

(ASK ALL UNLESS THEY REPORTED NO BETS AT E1 OR NO FREE BETTING INFO SERVICES AT FAS1a)

EFAS4. **In the last 48 hours**, how much was your betting **influenced** in the following ways by tips, information or messages from the **free betting info services** you use (pipe through names of free services from FAS1b)?

|  | Not at all | A little | A lot |
| --- | --- | --- | --- |
| How much I bet |  |  |  |
| My betting selections |  |  |  |
| The inducements I used |  |  |  |
| Which wagering operators I bet with |  |  |  |

ESGHS. **In the last 48 hours**, did you experience any of the following **as a result of your** **sports or race betting**? (Please select one response on each line)

|  | No | Yes |
| --- | --- | --- |
| Reduction of your available spending money |  |  |
| Less spending on recreational expenses such as eating out, going to the movies or other entertainment |  |  |
| Reduction of your savings |  |  |
| Sold personal items |  |  |
| Increased credit card debt |  |  |
| Had regrets that made you feel sorry about your betting |  |  |
| Felt like a failure |  |  |
| Felt ashamed of your betting |  |  |
| Felt distress about your betting |  |  |
| Spent less time with people you care about |  |  |

ENEXT1. **In the next 48 hours**, how many **bets** do you **intend** to place on sports and races?

(text box)

ENEXT2. **In the next 48 hours,** about how much **money** do you intend to **place** on sports and race bets?

Enter $

# (Instructions for forwarding messages)

ASK IF ANSWERED ANYTHING EXCEPT “0” EMAILS, TEXT MESSAGES AND NOTIFICATIONS RECEIVED AT EWO2 OR EPT2 OR EFAS2

**Please forward your direct messages to us**

We need you to please forward any emails, text messages and notifications to us that you received in the last 48 hours from wagering operators, paid tipsters and free betting info services. We’ve provided instructions for how to do this below.

Please try to delete any personal details in the messages before you send them to us. If you don’t delete them, we will replace any personal details with your unique participant code. Please **include this code (insert unique ID here)** when you forward messages to us. We take your anonymity seriously, and your name, email address or phone number will not be stored or reported.

**How to forward emails to us**

You noted you received (pipe from EWO2.1 + EPT2.1 + EFAS2.1) email(s) from wagering operators, paid tipsters and free betting info services in the last 48 hours. Please forward these emails to project email address and **be sure to include this unique ID (insert unique ID here).**

**How to forward text messages to us**

You noted you received (pipe from EWO2.2 + EPT2.2 + EFAS2.2) text messages from wagering operators, paid tipsters and free betting info services in the last 48 hours. Please forward these text messages to project phone number and **be sure to include this unique ID (insert unique ID here).**

For iOS (such as iPhone) hold your finger down on the message you'd like to forward. A pop up will appear. Please then select the “more” option. Click on the arrow which will appear at the bottom of the page. Then please enter project phone number, add your unique code and send.

For Android systems (such as Samsung phones), hold your finger down on the message, then a list of options will appear. Press “forward”, enter project phone number into the recipient box, add your unique code and send.

**How to forward notifications to us**

You noted you received (pipe from EWO2.3 + EPT2.3 + EFAS2.3) notifications from wagering operators, paid tipsters and free betting info services in the last 48 hours. Please forward these notifications to project email address or project phone number and **be sure to include this unique ID (insert unique ID here).**

App notifications typically appear on your phone, often on your home screen. The best way to forward a notification is to first take a screenshot.

For iOS (such as iPhone), if your device has a home button (the round button under the screen), press and hold the “power” button and press the “home” button. If you don’t have a home button, hold down the “power” button and the “volume up” button at the same time. You may hear a camera shutter noise and see a flash. The screenshot will appear and you will be able to save it to your photos so you can email or SMS it to us.

For Android devices, press the power and volume down buttons at the same time. If that doesn’t work, press and hold the power button and select screenshot. At the bottom left, you’ll find a preview of your screenshot. You can also find it in your Photos app. Tap “Library” then “Screenshots”. From there, you can edit and share it with us via email or SMS.

**Finally, please confirm …**

F1. Please confirm that you have forwarded to project email address OR project phone number the emails, text messages and notifications received in the last 48 hours from wagering operators, paid tipsters and free betting info services, and **have included this unique ID (insert unique ID here).**

- No, I have not
- Yes, I have forwarded messages to you

IF F1 = NO

We understand that we're asking you to do a bit by sending emails, messages and notifications.

Please remember that your number or email address will never be shown, and if the email or SMS or notification includes anything identifiable, this will also never be shown. It would be helpful if you could please share emails, messages or notifications with us, so that we can see what you are sent.

Thank you in advance for considering this.

(ASK IF ANSWERED “0” EMAILS, TEXT MESSAGES AND NOTIFICATIONS RECEIVED AT EWO2 AND EPT2 AND EFAS2)

**Finally, please confirm …**

You said you have not received any wagering emails, texts or notifications in the last 48 hours.

**But we still need to hear from you!** Please send an email to project email address OR text message project phone number saying “I have not received any messages from wagering operators, paid tipsters and free betting info services in the last 48 hours. Please **be sure to include this unique ID (insert unique ID here).**

Please click >> to move to the last page

If gambling is a problem for you or others, please call the Gambling Helpline on 1800 858 858 or go to [www.gamblinghelponline.org.au](http://www.gamblinghelponline.org.au) for free, confidential advice, available 24/7. If this questionnaire has raised any other issues for you, please call Lifeline on 13 11 14.

**Thanks for taking the time to complete the survey. We really appreciate your time and effort.**

**We’ll send you a link to the next short survey on [DAY] [DATE].**

**Remember, every survey you complete is valuable!**

**Please click** >> **to submit your responses.**
