# Supplementary figures and images for: Direct gambling marketing, direct harm: A randomised experiment
Source: Addiction. 2026 Mar 18;121(7):1907–19. doi: 10.1111/add.70369 (PMC13291081; doi:10.1111/add.70369)

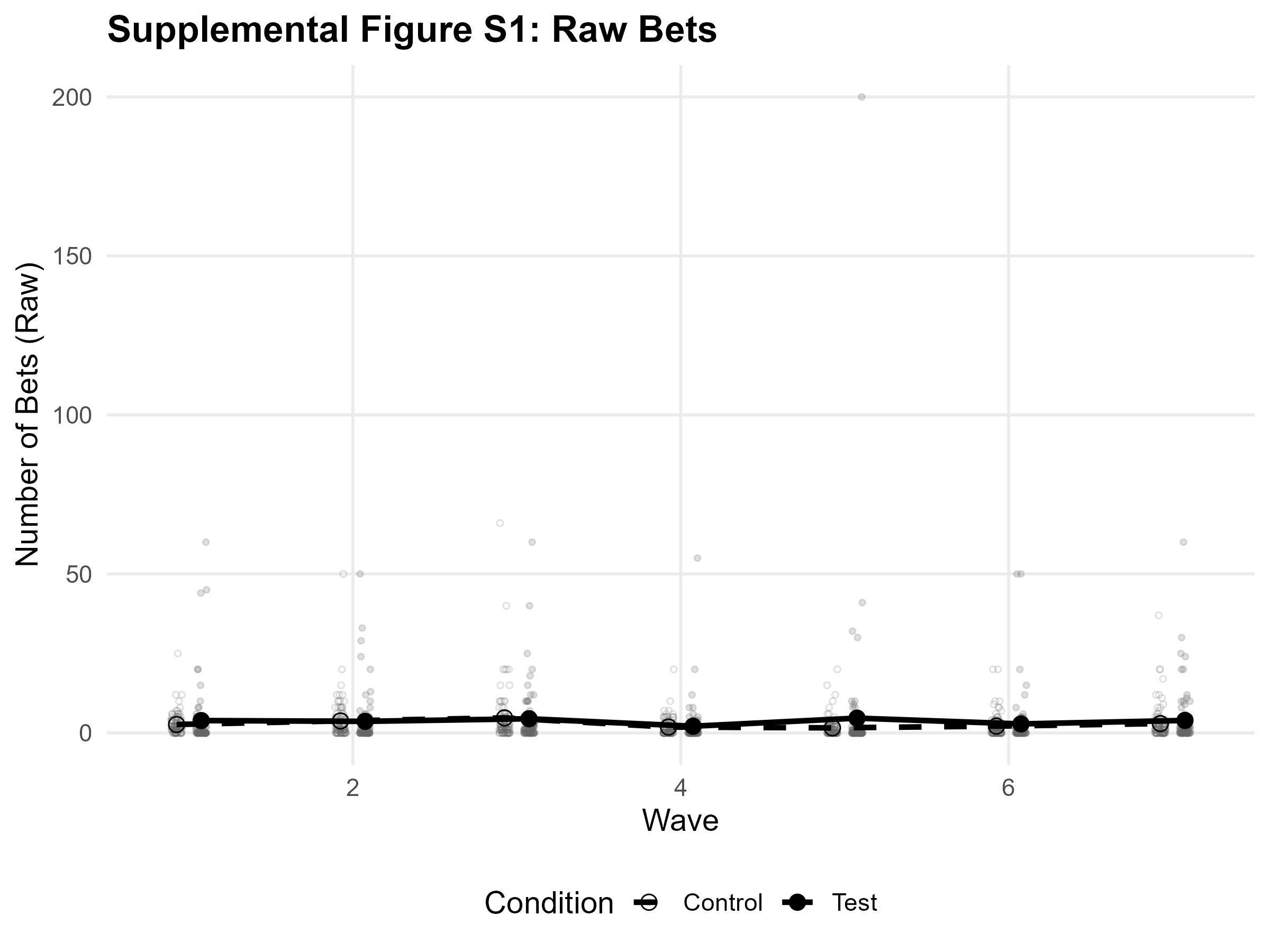

Supplement: Supplementary file 3 — Figure S1. Raw bets. [file ADD-121-1907-s003.png]

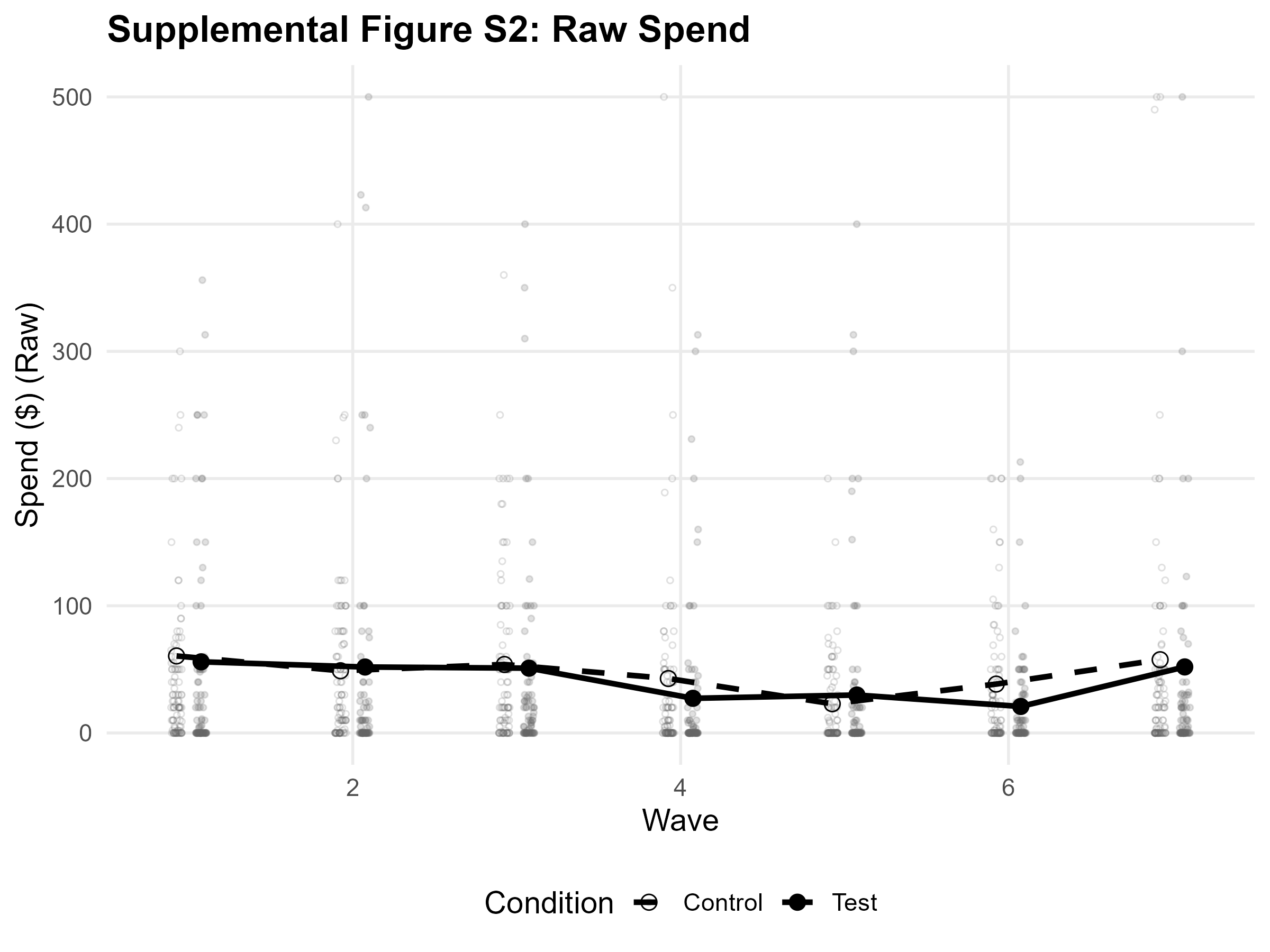

Supplement: Supplementary file 4 — Figure S2. Raw spend. [file ADD-121-1907-s002.png]

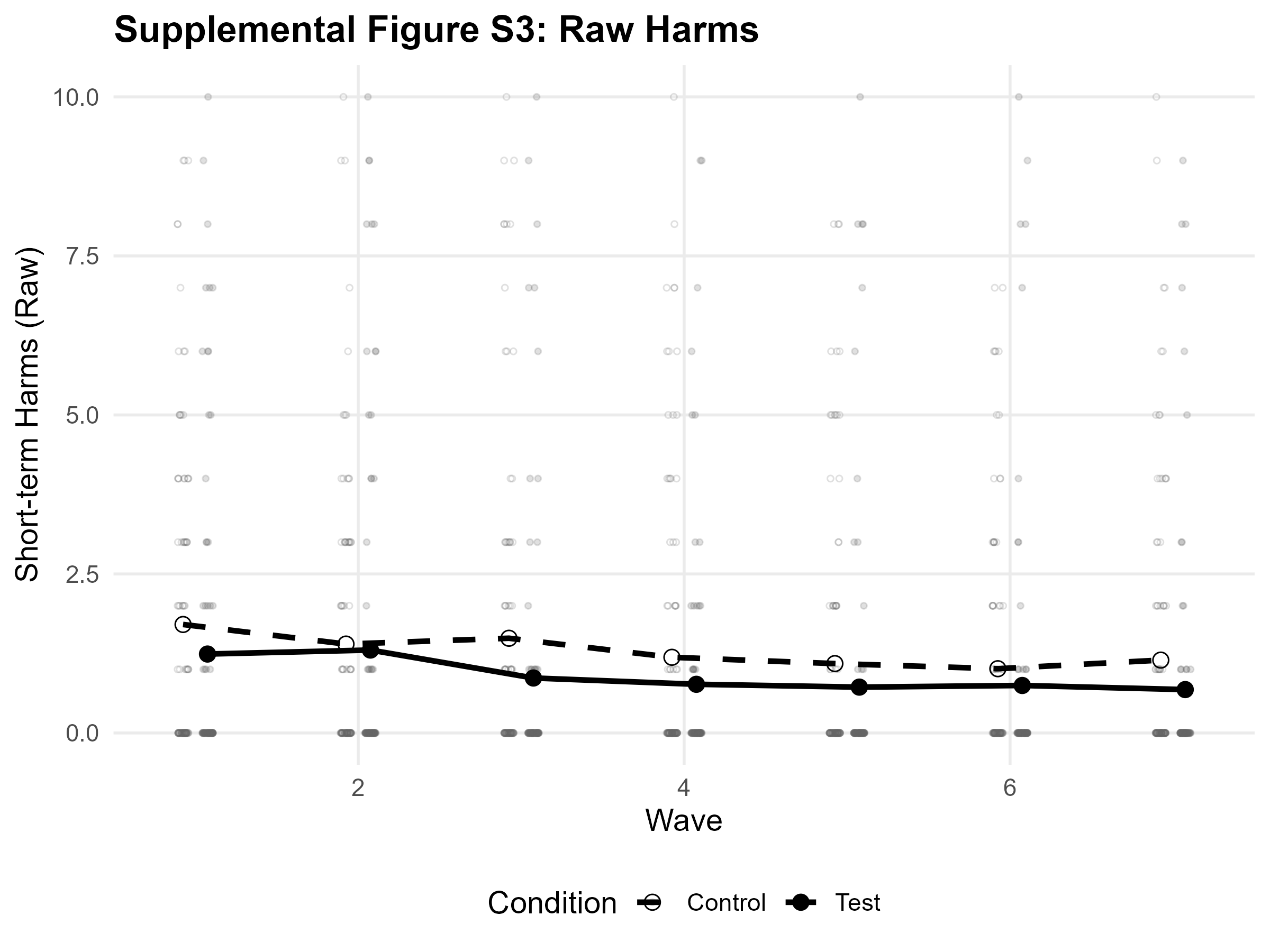

Supplement: Supplementary file 5 — Figure S3. Raw harms. [file ADD-121-1907-s005.png]
