# Supplementary material for: Direct gambling marketing, direct harm: A randomised experiment
Source: Addiction. 2026 Mar 18;121(7):1907–19. doi: 10.1111/add.70369 (PMC13291081; doi:10.1111/add.70369)
Supplement: Supplementary file 6 — Table S1. Baseline characteristics by eligibility in principle for the experiment (participants with complete data, N = 1,015). [file ADD-121-1907-s004.docx]

**Supplementary Table S1.** Baseline characteristics by eligibility in principle for the experiment (participants with complete data, N = 1,015)

| Characteristic | Category | Eligible for experiment  (n = 405) | Not eligible  (n = 610) |
| --- | --- | --- | --- |
| Age group | 18–39 years | 182 (44.9%) | 314 (51.5%) |
|  | 40+ years | 223 (55.1%) | 296 (48.5%) |
| Gender | Male / other | 225 (55.6%) | 373 (61.1%) |
|  | Female | 180 (44.4%) | 237 (38.9%) |
| PGSI category | Non-problem / low-risk | 180 (44.4%) | 278 (45.6%) |
|  | Moderate-risk / problem | 225 (55.6%) | 332 (54.4%) |
| Number of wagering operators | One operator | 170 (42.0%) | 237 (38.9%) |
|  | Two or more operators | 235 (58.0%) | 373 (61.1%) |

*Note.* “Eligible for experiment” is defined as meeting all the following baseline criteria: provided consent, reported ≥1 active wagering account, agreed to complete the EMA surveys, and agreed to opt out of direct marketing. Frequencies and percentages are based on participants with non-missing data on all listed variables (N=1,015); overall, 405/1,555 (26.0%) baseline respondents were eligible in principle.
